# Inflammatory Pseudotumor of the Liver Complicated by Lung Necrosis and Pleural Empyema: A Case Report

**DOI:** 10.1155/2011/504619

**Published:** 2011-09-06

**Authors:** Klaus Steinbrück, Marcelo Enne, Reinaldo Fernandes, Jose M. Martinho, Lúcio F. Pacheco-Moreira

**Affiliations:** Liver Transplantation Unit, Bonsucesso Federal Hospital—Health Ministry, Rio de Janeiro, Brazil

## Abstract

Inflammatory pseudotumor of the liver (IPTL) is a rare condition, but an important differential diagnosis of hepatic space-occupying lesions. It may regress spontaneously and mimic other liver tumors. Complications are usually intrahepatic. Herein, we present a case of IPTL which developed pleural empyema and lung necrosis as an uncommon complication.

## 1. Introduction

Inflammatory pseudotumor, also called inflammatory myofibroblastic tumor or plasma cell granuloma, xanthomatous pseudotumor, and inflammatory fibrosarcoma [1], is a rare condition. It may occur in various organs and is most commonly found in the lung, followed by the liver [2]. Inflammatory pseudotumor of the liver (IPTL) is a benign affection and an important differential diagnosis of space-occupying lesions of the liver. Although IPTL presents a good prognosis, associated complications have been described, like spontaneous rupture [3], portal vein thrombosis [4], and intrahepatic litihasis [5]. Herein, we present a case of IPTL complicated by pleural empyema and lung necrosis. To our knowledge, this is the first report of such complications related to IPTL.

## 2. Case Report

**Figure 1:**
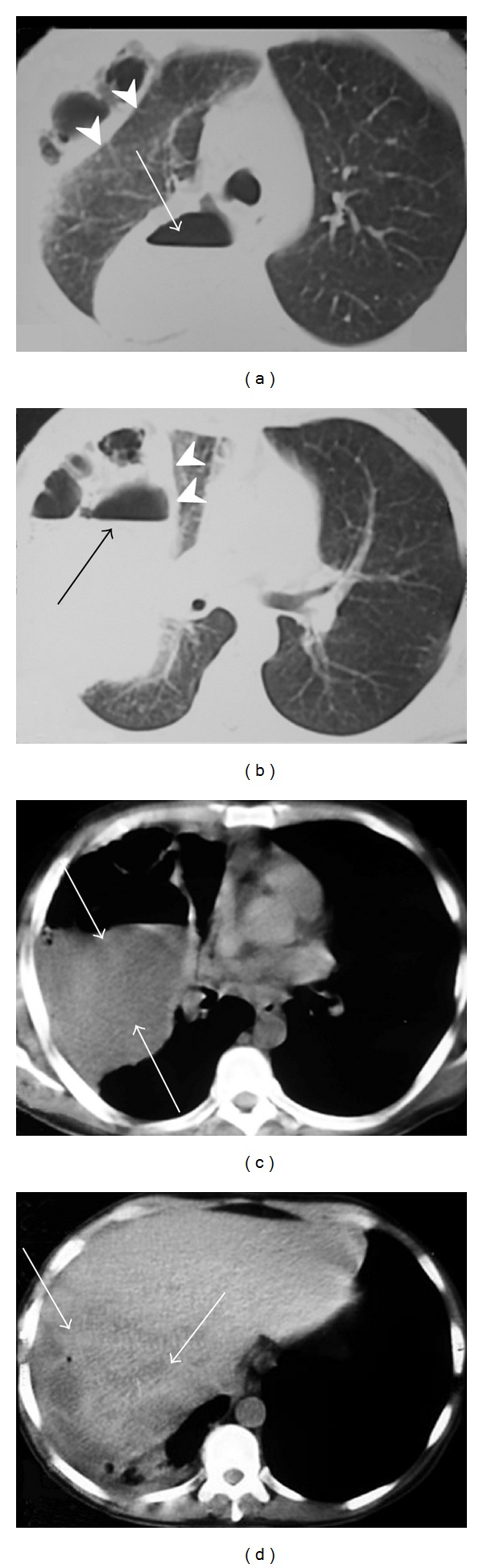
(a) and (b) computed tomography showing a collapsed right lung (arrow heads) and gas-fluid levels in the right hemithorax (arrows); (c) pleural effusion (arrows); (d) hepatic mass in segments VI and VII (arrows).

**Figure 2:**
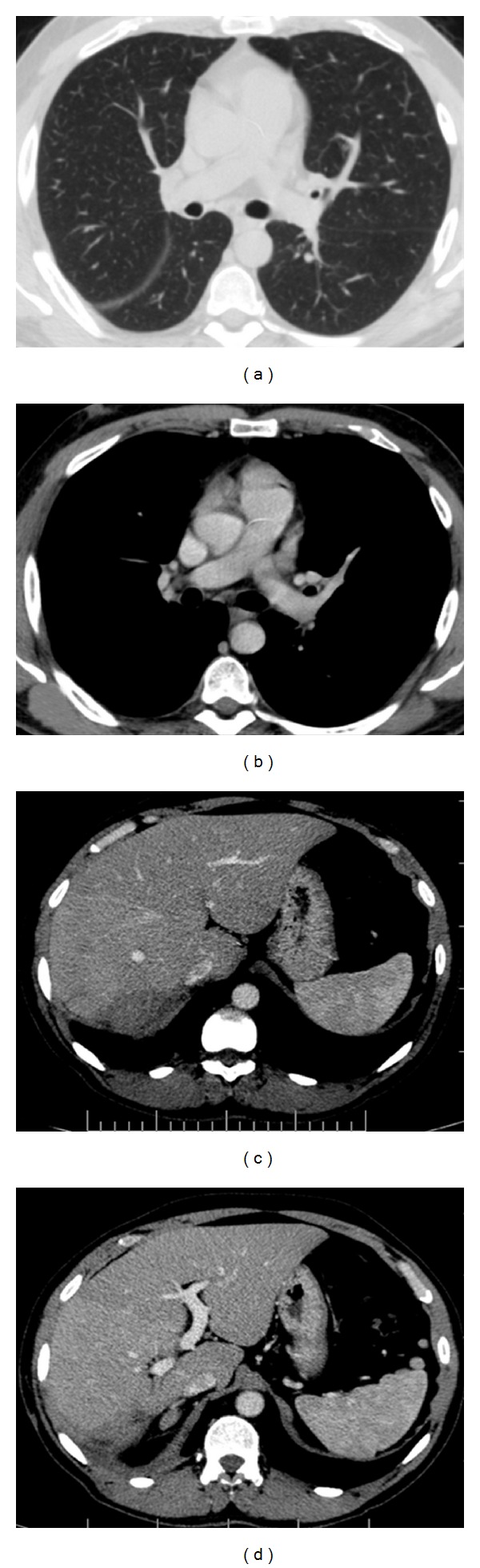
(a) and (b) computed tomography showing complete recovery of pulmonary lesions in right hemithorax; (c) and (d) almost complete disappearance of hepatic lesion (arrows).

A 47-year-old man was referred to our institution with a 2-month history of jaundice, weight loss, and intermittent low fever. On admission, physical examination showed a tender mass in the right-upper quadrant. A previous computed tomography (CT) revealed a mass of 9.0 cm in segments VI and VII of the liver, with contrast enhancement in arterial phase and washout at equilibrium phase, suggestive of hepatocellular carcinoma. Colonoscopy and upper endoscopy were normal. Serology was negative for hepatitis B and C. Alpha-fetoprotein was 2.3 ng/dL. Other laboratory tests revealed leucocytosis (12,800 cell/mm^3^) and elevated C-reactive protein (CRP) (5.2 mg/dL). Percutaneous liver biopsy with tru-cut needle, under ultrasound guidance, was performed and demonstrated hepatic tissue with proliferation of spindle-shaped cells mixed with inflammatory myo-fibrohistiocytes, suggesting IPTL diagnosis. Right hepatectomy was indicated, but before patient was admitted for surgery, he developed severe dyspnea and general fatigue. Thorax and abdominal CT (Figure 1) showed a pleural effusion in right hemithorax associated to pleural thickness, gas-fluid levels, and a collapsed right lung. Laboratory tests demonstrated high leucocytosis (21,500 cell/mm^3^) and rise of CRP (17.7 mg/dL). Antibiotics were initiated, and an exploratory thoracotomy was performed, which revealed a bulky pleural empyema (approximately two liters), right lung's lower lobe necrosis, a portion of right diaphragm destruction, and a necrosed cavity in right liver. A lower lobectomy of the right lung was executed in addition to diaphragm suture and pleural space cleaning and drainage. Patient presented a satisfactory recovery after surgery. Pleural drain was removed on postoperative day (POD) 9, and patient was discharged on POD 18. Histopathological analysis of the resected lung demonstrated pneumonitis with necrosis and fibro-anthracosis. CT images five months after surgery (Figure 2) showed complete recovery of pulmonary lesions and almost complete disappearance of hepatic lesion. Patient is well and asymptomatic nine months after surgery.

## 3. Discussion

Since it was first described by Pack and Baker in 1953 [6], IPTL has been more reported, probably due to development in imaging procedures. It is considered to be a benign reactive inflammation condition with unclear pathogenesis [7]. Even though IPTL may regress spontaneously or following antibiotic treatment, surgical resection still is applied as main treatment option, especially for patients with severe symptoms or uncertain diagnosis [3, 4]. Clinical and radiologic features of IPTL may mimic other liver conditions like hepatocellular carcinoma (HCC) [4], cholangiocarcinoma [8], liver metastasis [4], or liver abscess [4], which lead to surgical approach. 

Surgical complications described in association to IPTL are mostly locally intrahepatic events. Tang et al. [3] reported two cases of spontaneous rupture of IPTL treated by segmental hepatectomy. Kai et al. [8] reported a case of left hepatic vein thrombosis and Arantius' ligament infiltration associated with multiple IPTL imitating cholangiocarcinoma. Tsou et al. [4] described a case of IPTL mimicking HCC with left portal vein thrombosis. Ueda et al. [5] related a case of IPTL causing segmental bile duct obstruction to intra-hepatic stone formation.

Here, we present a case of extrahepatic, extraabdominal complication related to IPTL. In this case, the patient developed a severe inflammatory involvement of diaphragm, pleura, and lung. We believe that a spontaneous rupture of the liver mass occurred, as it was in close contact to the diaphragm, the inflammatory process spread to the right thoracic cavity, leading to segmental destruction of the diaphragm, pleural empyema, and necrosis of right lung's lower lobe. The patient was treated through thoracotomy. Laparotomy was not necessary, once the liver was firmly adhered to the diaphragm and no contamination of the abdominal cavity occurred. Follow-up imaging examination revealed a good outcome for the thoracic lesions and almost total regression of the liver mass.

In conclusion, inflammatory pseudotumor is a differential diagnosis for liver tumors. Even though IPTL may regress spontaneously or after antibiotic treatment, it can evolve to serious intra- or extrahepatic complications. To the best of our knowledge, this is the first report of pleural empyema and lung necrosis as complications of IPTL.
